# Structural dynamics of LaVO_3_ on the nanosecond time scale

**DOI:** 10.1063/1.5045704

**Published:** 2019-01-18

**Authors:** Matthew Brahlek, Vladimir A. Stoica, Jason Lapano, Lei Zhang, Hirofumi Akamatsu, I-Cheng Tung, Venkatraman Gopalan, Donald A. Walko, Haidan Wen, John W. Freeland, Roman Engel-Herbert

**Affiliations:** 1 Department of Materials Science and Engineering, Pennsylvania State University, University Park, Pennsylvania 16801, USA; 2 Advanced Photon Source, Argonne National Laboratory, Argonne, Illinois 60439, USA; 3 Materials Research Institute, Pennsylvania State University, University Park, Pennsylvania 16801, USA; 4 Department of Physics, Pennsylvania State University, University Park, Pennsylvania 16801, USA; 5 Department of Engineering Science and Mechanics, Pennsylvania State University, University Park, Pennsylvania 16801, USA; 6 Department of Chemistry, Pennsylvania State University, University Park, Pennsylvania 16801, USA

## Abstract

Due to the strong dependence of electronic properties on the local bonding environment, a full characterization of the structural dynamics in ultrafast experiments is critical. Here, we report the dynamics and structural refinement at nanosecond time scales of a perovskite thin film by combining optical excitation with time-resolved X-ray diffraction. This is achieved by monitoring the temporal response of both integer and half-integer diffraction peaks of LaVO_3_ in response to an above-band-gap 800 nm pump pulse. We find that the lattice expands by 0.1% out of plane, and the relaxation is characterized by a biexponential decay with 2 and 12 ns time scales. We analyze the relative intensity change in half-integer peaks and show that the distortions to the substructure are small: the oxygen octahedral rotation angles decrease by ∼0.3° and La displacements decrease by ∼0.2 pm, which directly corresponds to an ∼0.8° increase in the V-O-V bond-angles, an in-plane V-O bond length reduction of ∼0.3 pm, and an unchanged out-of-plane bond length. This demonstration of tracking the atomic positions in a pump-probe experiment provides experimentally accessible values for structural and electronic tunability in this class of materials and will stimulate future experiments.

Transition metal oxides (TMOs) with a perovskite structure show a wide range of phenomena from metal-insulator transitions and novel magnetic phases to high-temperature superconductors; all these are closely related to the strong electron-electron interactions of the neighboring transition metal cations and, thus, to the local bonding environment.^1^ Therefore, targeting structural modification holds great promise for controlling the global electronic phase, which has been borne-out in epitaxial strained perovskite thin films,^2^ where changes to the local bonding geometry can induce ferroelectricity,^3^ magnetism,^4^ or both,^5^ as well as modulating the critical superconducting temperature.^6^ In particular, dynamic manipulation of the structure on the ultrafast time scale can drive non-equilibrium changes to the electronic ground state and enable accessing otherwise hidden, metastable ground states.^7–11^

The TMO perovskites have the *AB*O_3_ formula unit, where *A* is typically a group II alkali-earth or a rare-earth element and *B* is a transition metal element. The perovskite structure is characterized by a cubic unit cell consisting of the *A*-site cation at the corners and oxygen atoms at the face centers which form an oxygen octahedron enclosing the *B*-site cation at its center. As shown in Fig. 1(a), the basic structure can be selectively deformed to accommodate many combinations of cations with different ionic radii without losing the corner connectivity to adjacent octahedra; this is accomplished through specific rotations of the oxygen octahedron by angles *α*, *β*, or *γ* about the pseudocubic *x*, *y*, or *z* axes, respectively.^12–16^ For coherently strained LaVO_3_ (space group *Pbnm*) grown epitaxially on SrTiO_3_ (001), the compressive strain favors the orthorhombic *c*-axis to be in the film plane,^17^ which can be described simply using Glazer notation^13,18^ as *a^−^a^+^c*. This indicates that the lattice parameters and octahedral rotation angles along the pseudocubic *x*- and *y*-axes are the same (lattice parameter = *a* and *α* = *β*), with an out-of-phase rotation along the *x*-axis (the *–* superscript) and an in-phase rotation along the *y*-axis (the + superscript); while along the pseudocubic *z*-axis (normal to the film surface), the lattice parameter is different with an out-of-phase rotation angle *γ*, shown in Figs. 1(c) and 1(d).^17^ Rotations of the oxygen octahedra are particularly important because they are directly related to the *B*-O-*B* bond angle and *B*-O bond length, thus affecting the atomic coupling strength between neighboring *B*-site cations and thus the electronic bandwidth.^19^ Since a large out-of-plane expansion can be induced in epitaxial thin films, while not affecting the in-plane lattice parameters, as dictated by the substrate which is typically chosen to not directly adsorb at the pump wavelength, ultrafast excitation can be used to uniquely modify rotations of the oxygen octahedra in a way that does not follow the static thermal trajectory that is accessible by changing the temperature of the film and the substrate; thus, this can modulate electronic properties and may be used to search for entirely new metastable phases.

**FIG. 1.**
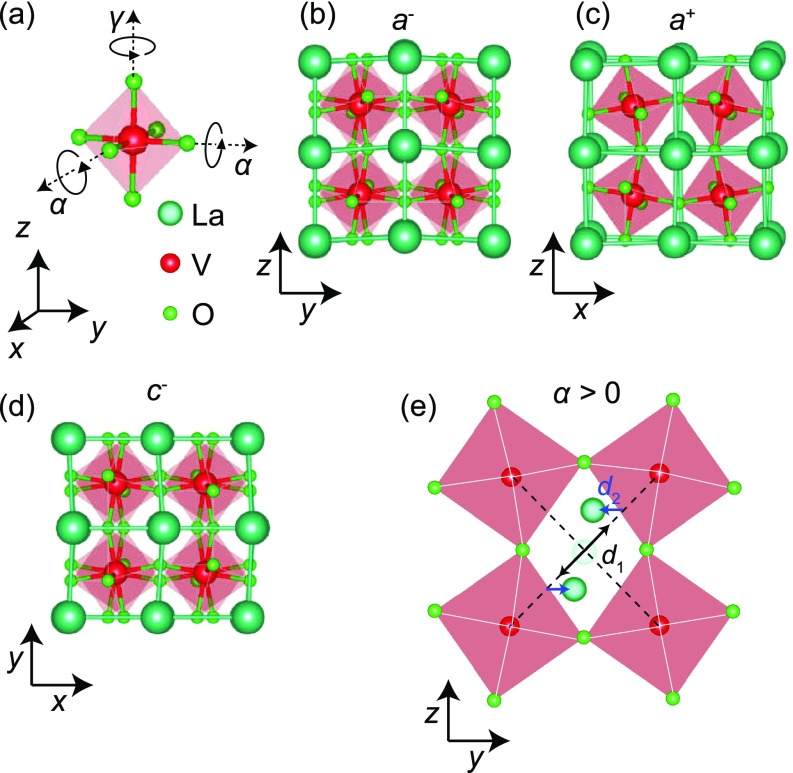
(a) The *a*^−^*a*^+^*c*^−^ structure of LaVO_3_ characterized by rotations of an oxygen octahedron about the pseudocubic *x*, *y*, and *z* axes by the respective angles of *α*, *α*, and *γ.* Projection of the doubled unit cell along the (b) *x*, (c) *y*, and (d) *z* axes, with out-of-phase and in-phase rotation patterns of the oxygen octahedra along *x, z*, and *y*, respectively. (e) The La atoms displace perpendicular to the + axis in a pattern that doubles the periodicity of the pseudocubic unit cell and is quantified by the parameters *d*_1_ and *d*_2_.

Direct quantification of octahedral angles is achieved by measuring the intensity of a specific set of half-order diffraction peaks that originate from structural distortions resulting in a doubling of the pseudocubic unit cell along the out-of-phase axes.^13–15,18^ In contrast to bulk crystals, the extraction of the individual parameters associated with these structural distortions is particularly challenging in thin films due to the small scattering cross-section of the oxygen atoms as well as the small scattering volume, thus requiring a high brilliance X-ray source.^17,20–22^ In addition to rotations, any distortion that doubles the unit cell will contribute to the intensity of the half-order peaks, including *A*-site cation displacements, breathing modes, etc. As shown in Fig. 1(e), for *Pbnm*-type perovskites, the dominate distortion other than oxygen octahedral rotations occurs at the *A*-sites where the cations displace out of their high symmetry position perpendicular to the in-phase rotation axis. This results in a complex convolution of the different contributions to their total intensity.^23^ Further, tracking the dynamic response in a pump-probe experiment adds an additional challenge because achieving the temporal resolution requires a ∼1000× reduction in the integration time and thus a reduced signal.

Despite these challenges, we report a structural refinement of thin films of LaVO_3_, a Mott insulator with a bandgap of ∼1.1 eV,^24^ on the nanosecond time scale when pumped by above-band-gap 800 nm (1.55 eV), 50 fs light pulses. This was achieved with a full structural factor calculation using a geometric model of the atomic positions which only captures dominate distortions in LaVO_3_, namely, the rotation of the oxygen octahedra and La displacements.^25^ We found that the unit cell of the LaVO_3_ film responded to the pump pulse at 100 ps by a ∼0.1% (∼0.3 pm) expansion along the out-of-plane direction and relaxation followed a biexponential with two different time constants. The structural refinement procedure revealed that in response to the pump pulse, the oxygen octahedral angles as well as the *A*-site displacements decreased, and thus, the V-O-V bond angle increased, and the in-plane V-O bond length decreased, while the out-of-plane bond length was static. This is consistent with the film being pushed towards the high temperature, high symmetry cubic phase. This demonstrated that it is possible to track the response of rotation angles and *A*-site displacements in ultrathin oxide thin films, now allowing for a comprehensive structural refinement and thus for a full structural-electronic trajectory to be mapped at ultrafast time scales.

To resolve the structural dynamics, ultrafast X-ray diffraction experiments were performed at the Advanced Photon Source at 7ID-C beamline. LaVO_3_ films 50 nm thick were grown on SrTiO_3_ substrates by the *hybrid* molecular beam epitaxy technique (see supplementary material).^26,27^ A monochromatic 11 keV X-ray beam incident on the sample and the diffracted beam were detected by a gated single-photon-counting area detector (Pilatus 100K).^28^ The measurements were performed using a 6-circle goniometer with the fixed incident angle of 10°, while the laser was maintained near normal incidence. To account for variations of the incident synchrotron X-ray beam, flux measurements of the main beam were made upstream of the sample with an ion chamber, and the measured intensities were normalized to this signal. The temporal resolution of the X-ray diffraction experiment was limited by the electron-bunch temporal width to ∼100 ps. A 60 fs Ti:sapphire laser (800 nm) with a repetition rate of 1 kHz was used to optically excite the LaVO_3_ film. The incident laser fluence was fixed at ∼75 mJ/cm^2^, which was chosen to give a sufficient response, while being significantly below the damage threshold. The laser's adjustable time delay was phase-locked relative to the synchrotron X-ray pulse. Kirkpatrick-Baez mirrors were used to focus the X-ray beam down to a spot size of ∼50 *μ*m at the center of the much larger ∼600 *μ*m × 1000 *μ*m laser spot, which ensured that the probed volume was homogeneously excited.

Figure 2(a) shows *θ*-scans (rocking curves) before and after the pulse arrived. There are two main features that are discernible from this response (1) the small decrease in peak intensity and (2) the shift of the peak position; this was quantified by a Gaussian to the peaks, shown as solid lines in Fig. 2(a), which enabled the extraction of the peak position and intensity. The first feature to note is the decrease in peak intensity right after the optical excitation, which at around a delay of 0.6 ns was about 6%. This is consistent with the thermal-induced increase in incoherent scattering caused by the motion about the equilibrium position of atoms.^29,30^ The second feature to note is the shift in the X-ray peak to lower *θ* after the pulse and hence 2*θ*, as shown in Fig. 2(b), which indicates an expansion of the lattice. This corresponds to a maximum of 0.3 pm (0.1%) out-of-plane expansion of the film due to a temperature increase. This allows for a temperature rise *ΔT* to be estimated using the thermal expansion coefficient (*Δa*/*a*_0_)/(*ΔT*) ≈ 1–2 × 10^−5^/K,^31^ where *Δa* is the change in the lattice parameter and *a*_0_ is the unit cell parameter prior to excitation (3.9507 Å). In this expression, *Δa* was corrected for the Poisson ratio (∼0.3–0.4)^32^ to account for the static in-plane lattice parameter due to the epitaxial relation to the substrate. The estimated change in temperature is then *ΔT* ≈ 20–50 K. For comparison, we can independently estimate the temperature rise of the film with the incident pulse using the known adsorption properties of LaVO_3_. The energy adsorbed per pulse^33^ by the LaVO_3_ film is given by ${Q=F}_{\text{incident}}\frac{\left(E_{p}-E_{g}\right)}{E_{p}}\left(1-R\right)\frac{e^{-\frac{t}{\zeta }}}{t}$, assuming a dominant radiative decay of electron-hole pairs (reasonable for the nanosecond time scales used here), where *F_incident_* ≈ 75 mJ/cm^2^ is the incident laser fluence, *E_p_* is the pump energy, *E_g_* is the bandgap of LaVO_3_,^24^ *R* ≈ 0.22 is the reflectivity at 800 nm,^34^ and *ζ* ≈ 1000 nm is the adsorption length at 800 nm.^35^ The difference in adsorption near the surface in comparison to that near the film-substrate interface is within a few percent, which is the result of the thickness being much smaller than *ζ*. Therefore, the approximation that the film is homogeneously excited can be made. This yields an average temperature rise *ΔT* ≈ *Q/C*, where *C* ≈ 2.8 J/(cm^3^ K) is the heat capacity of LaVO_3_ at room temperature.^36^ From this calculation, it is found that a temperature rise of around 50 K is estimated, which is in reasonable agreement with the estimation from the observed change in the lattice parameter.

**FIG. 2.**
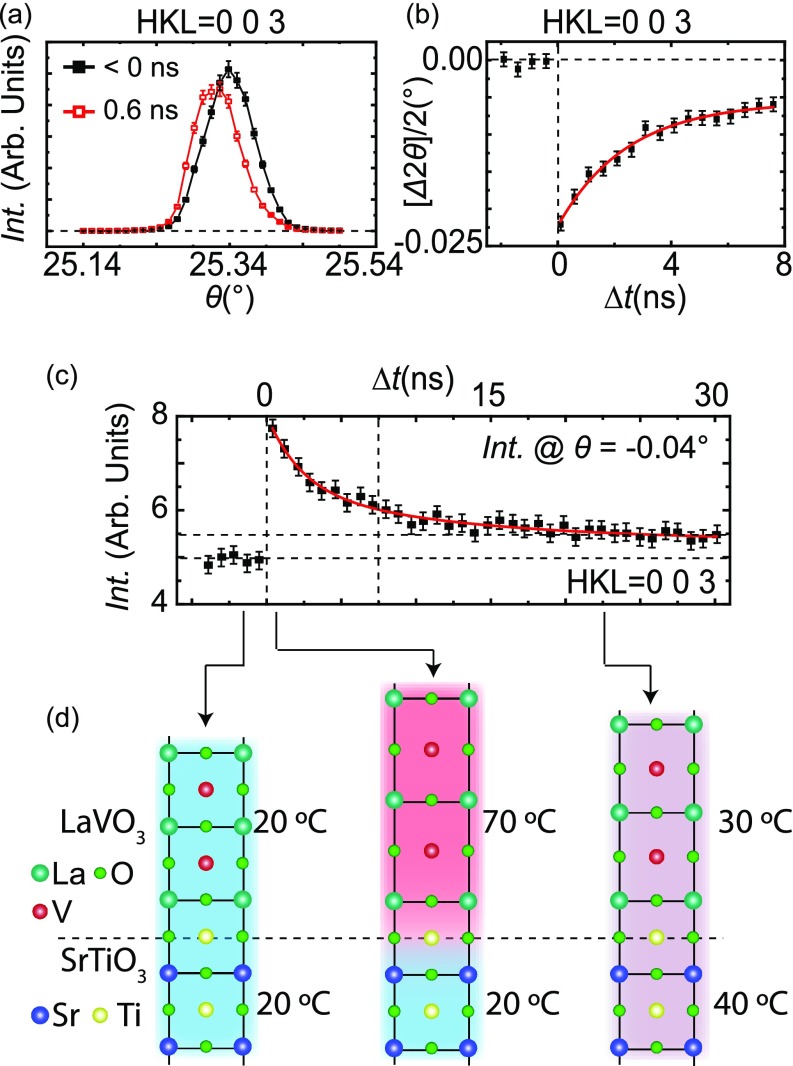
(a) *θ*-scans before (black filled squares) and 0.6 ns after (red open squares) laser excitation. The symbols are the data, and the solid line is a Gaussian fit to the data. (b) The change in the peak position of the *θ*-scans in (a) as a function time delay which gives the change in 2*θ*. (c) The intensity measured at the full-width half-max position of the *θ*-scans as a function of time delay. (d) Schematic representation of the unit cell dynamics extracted from the change in the peak position of the *θ*-scans in (a), which, as discussed in the text, can be used to deduce the indicated time-dependent temperature of the LaVO_3_ film and interfacial layers of the SrTiO_3_ substrate.

The temporal relaxation was determined from two different parameters: (1) the peak position as a function of time delay was measured by performing *θ*-scans of the (0 0 3) peak as shown in Fig. 2(b) and (2) by fixing the *θ* position at the full-width half maximum, i.e., −0.04° off the position of the maximum intensity, and measuring the intensity while varying the delay time, shown in Fig. 2(c). It can be seen in Fig. 2(b) that the lattice expansion occurred within the 100 ps resolution of the X-ray pulse, which is consistent with the fast creation of electron-hole pairs from the above bandgap excitation and fast energy relaxation to the band edges through the generation of phonons. It can be further seen that the initial cooling of LaVO_3_ occurred quickly, which was followed by a slower decay. From the data in Figs. 2(b) and 2(c), it is found that the fast relaxation, *τ*_1_, can be seen to occur with a few nanoseconds. The longer time decay, *τ*_2_, can be more clearly seen in the delay scans in Fig. 2(c). Here, the lattice has not relaxed back to the undisturbed, initial state even after ∼30 ns. We fit these data to a biexponential decay^33^ and confirmed that the fast relaxation time constant was *τ*_1_ ≈ 2 ns, while the longer time constant was around *τ_2_* ≈ 12 ns. The origin for this biexponential decay can be attributed to the process by which the LaVO_3_ film conducted heat into the SrTiO_3_ substrate.^33^ As schematically shown in Fig. 2(d), the laser pulse was only adsorbed by LaVO_3_ since the bandgap of the SrTiO_3_ (∼3.25 eV = 380 nm) is much larger than the photon energy of the laser. The heat generated by the absorption in LaVO_3_ quickly transfers from the film to the substrate, heating up the colder SrTiO_3_ in the vicinity of the film-substrate interface. The thermal conduction is now reduced, slowing down the heat transfer out of LaVO_3_, which accounts for the longer time scale.

With the dynamics of the unit cell established, we now move onto refining the atom positions by comparing the relative intensity change in the selected half-order diffraction peaks. Following the procedure described in Ref. 23 and detailed in Ref. 26, specific half-order diffraction peaks, sensitive to the octahedral rotation angles as well as the La displacement pattern, were tracked, thus enabling a structural refinement. Figures 3(a)–3(e) show the intensity versus time, both experimental data shown as red, open circles and exponential fitting using the biexponential model discussed previously as black, filled squares. There are distinct differences between the change in the intensity of the half-order peaks shown in Figs. 3(b)–3(e) and the integer (1 0 2) and (0 0 3) peaks shown in Figs. 3(a) and 2(a), respectively. The direct comparison of these peak intensities revealed that the decrease was much larger for the half-order peaks and much smaller for the integer peaks; this reflects the changes in the atomic positions and hence bond angles and bond lengths, within the unit cell, and will be discussed next.

**FIG. 3.**
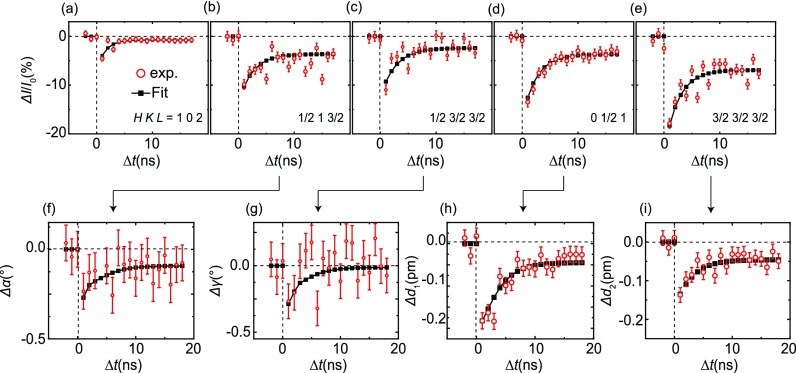
(a)–(e) The time dependence of the change in integrated intensity from an integer peak (1 0 2) (a) and selected half-order peaks (b)–(e). (f)–(i) The change in intensities of the peaks in (a)–(e) can be used to determine the change in the oxygen-octahedral rotation angles *α* (f) and *γ* (g), as well as the La-displacement parameters *d*_1_ (h) and *d*_2_ (i) (see the text for discussion of the error bars). Red open symbols are raw experimental data, while black filled squares are data fit to an exponential function (see the text).

Figures 3(f)–3(i) summarize the derived change in the structural parameters after the excitation pulse. All parameters decreased: *Δα* ≈ −0.3°, *Δγ* ≈ −0.3°, *Δd*_1_ ≈ *–*0.18 pm, and *Δd*_2_ ≈ *–*0.14 pm. The data shown in Figs. 3(f)–3(i) were obtained by fitting the change in X-ray peak intensities shown in Figs. 3(a)–3(e). Red, open circles in Figs. 3(f)–3(i) correspond to fitting the raw data in Figs. 3(a)–3(e), while the black, filled squares in Figs. 3(f)–3(i) correspond to fitting the biexponential-fitted data in Figs. 3(a)–3(e). By comparing the signal-to-noise ratio of the X-ray peak intensities used to extract the structural distortion parameters, the ($\frac{1}{2}1\frac{3}{2}$) and ($\frac{1}{2}\frac{3}{2}\frac{3}{2}$) reflections were found to have more variation than the ($0\frac{1}{2}1)\;$ and ($\frac{3}{2}\frac{3}{2}\frac{3}{2}$) peaks, resulting in higher noise levels seen in the rotation angles *α* and particularly *γ*, compared to the displacements *d*_1_ and *d*_2_. The higher level of noise in *γ* than *α* stems from non-linear error propagation in the numeric fitting procedure. To estimate the uncertainty in the angles, the error bars shown in Figs. 3(f)–3(i) were calculated by taking the standard deviation of the difference between the raw data (red, open circles) and the fitted data (black, filled squares), giving *Δα* ≈ −0.30° ± 0.10°, *Δγ* ≈ −0.30° ± 0.13°, *Δd_1_* ≈ −0.18 pm ± 0.02 pm, and *Δd_2_* ≈ −0.14 pm ± 0.02 pm; this change in the rotation angle corresponds to an increase in both the in-plane and out-of-plane V-O-V bond angles of *θ_ab_* ≈ *θ_c_* ≈ 0.8°, respectively, while the V-O bond lengths decrease in-plane by *Δd_ab_* ≈ 0.3 pm, and, interestingly, the out-of-plane bond length, *d_c_*, is found to be nearly unchanged, which can be rationalized by the counteracting effects of out-of-plane expansion (increasing the bond length) and the reduction in *α* (acting to shorten the bond length).

Several observations from the change in the structural parameters can be made: (1) The observation that the parameters decreased with laser excitation indicates that the crystal structure was being pushed towards a high temperature, high symmetry cubic phase. In fact, to accommodate the observed ∼0.1% out-of-plane expansion, for the ridge rotation model, it is expected that *α* should decrease, while *γ* should not change since the in-plane lattice parameters do not change. Nevertheless, a decrease in *α* and *γ* was observed, which indicates that the main driver behind the structural distortion is solely thermal. (2) This experiment provides realistic bounds for the magnitude of structural distortion in functional perovskite oxides in pump-probe experiments, with the conclusion that the values are modest. With recent focus on tailoring material properties based on modulating octahedral angles,^12^ this result will help guide theory to identify systems that are in close enough proximity to phase instabilities and only require a change in the structure to study the dynamics of electronic phase transitions.

In conclusion, we have performed a structural refinement of a perovskite film and thus demonstrated that it is possible to directly resolve all the atomic positions in response to optical excitation on nanosecond time scales. The film was found to undergo a ∼0.1% out-of-plane lattice expansion and decayed back to the initial structure via a biexponential heat transfer process into the substrate. Monitoring the dynamic response of selected half-order peaks enabled the refinement of both the oxygen octahedral angles, *α* and *γ*, which decreased by ∼0.3°, as well as the La displacements that also decreased by ∼0.2 pm. These modest distortions were correlated with an increase in the V-O-V bond angle by 0.8°, while the in-plane V-O bond length decreased by 0.3 pm and the out-of-plane bond length remained static. Unraveling the origins of non-equilibrium transient states necessitates the ability to completely refine changes in the perovskite structure on the pico- and femtosecond time scales, and the current work is a significant step towards this realization.

See supplementary material for additional information on thin film growth, static X-ray characterization, and the extraction of rotation angles and *A*-site displacements.
